# A Cross-Sectional Study of Experiences and Attitudes towards Clinical Audit of Farm Animal Veterinary Surgeons in the United Kingdom

**DOI:** 10.3390/vetsci5040084

**Published:** 2018-09-28

**Authors:** Katie Waine, Rachel S. Dean, Chris Hudson, Jonathan Huxley, Marnie L. Brennan

**Affiliations:** 1 Centre for Evidence-based Veterinary Medicine, School of Veterinary Medicine and Science, University of Nottingham, Sutton Bonington Campus, College Road, Leicestershire LE12 5RD, UK; katie.waine1@nottingham.ac.uk (K.W.); rachel.dean@vetpartners.com.uk (R.S.D.); 2 School of Veterinary Medicine and Science, University of Nottingham, Sutton Bonington Campus, College Road, Leicestershire LE12 5RD, UK; chris.hudson@nottingham.ac.uk (C.H.); j.huxley@massey.ac.nz (J.H.)

**Keywords:** clinical audit, farm animal veterinary surgeons, quality improvement, experiences, attitudes, questionnaire, survey

## Abstract

Clinical audit is a quality improvement tool used to assess and improve the clinical services provided to patients. This is the first study to investigate the extent to which clinical audit is understood and utilised in farm animal veterinary practice. A cross-sectional study to collect experiences and attitudes of farm animal veterinary surgeons in the UK towards clinical audit was conducted using an online nationwide survey. The survey revealed that whilst just under three-quarters (*n* = 237/325; 73%) of responding veterinary surgeons had heard of clinical audit, nearly 50% (*n* = 148/301) had never been involved in a clinical audit of any species. The participants’ knowledge of what a clinical audit was varied substantially, with many respondents reporting not receiving training on clinical audit at the undergraduate or postgraduate level. Respondents that had participated in a clinical audit suggested that protected time away from clinical work was required for the process to be completed successfully. This novel study suggests that clinical audit is undertaken to some extent in farm animal practice and that practitioner perception is that it can bring benefits, but was felt that more resources and support were needed for it to be implemented successfully on a wider scale.

## 1. Introduction

Clinical audit is a quality improvement tool used to assess and improve the clinical services provided to patients [1,2,3]. It involves the collection of data prospectively or retrospectively in health care settings to answer a specific question relating to the delivery of clinical care. The ultimate aim of clinical audit should be to improve the care delivered to patients and the service delivered to clients. Despite the increasing emphasis put on the process in the UK by the Royal College of Veterinary Surgeons (RCVS) [4], there have been no studies investigating the extent to which clinical audit is utilised in farm animal veterinary practice. Viner [5] previously surveyed the attitudes of veterinary surgeons towards clinical audit, but analysis was not carried out looking specifically at farm animal practice. Opinions on clinical audit were collected via interviews and the study was limited by a small sample size with only two veterinary surgeons representing rural practice [5].

In the medical profession, a number of studies have been conducted to collect thoughts about clinical audit, but have specifically focused on junior doctors as this group is commonly expected to facilitate clinical audits [6,7,8]. Barriers and facilitators to clinical audit have been highlighted, as well as the difficulties and benefits of the process, which may be applicable to veterinary practice [9]. These include practitioners not conducting clinical audit despite it being introduced as a concept [10] and consideration of the impact of the attitudes of the professionals expected to undertake the clinical audit on its success [9]. The benefits of focusing on the concerns of practitioners and improving those areas of concern, rather than focusing on clinical audit implementation methods themselves, have also been highlighted [11]. In small animal veterinary practice, time has been highlighted as a potential barrier to clinical audit, while good communication aids facilitation [2,5]. For clinical audit regulation and guidance to be successfully established and implemented in farm animal veterinary practice, it is important that the understanding of and current use of clinical audit by farm animal veterinary surgeons is considered. Therefore, the aim of this study were to gather experiences and attitudes of veterinary surgeons undertaking farm animal work in the UK towards clinical audit.

## 2. Materials and Methods

A cross-sectional study surveying the opinions of veterinary surgeons who undertake farm animal work in the UK was conducted through distribution of an online questionnaire.

### 2.1. Population of Interest

The target population was RCVS registered veterinary surgeons who worked in farm animal veterinary practice or undertook farm animal clinical work in the UK. The sampling frame used for the study was membership of the British Cattle Veterinary Association (BCVA), the Sheep Veterinary Society (SVS) and practices registered as carrying out clinical work for sheep and cattle on the RCVS “Find a Vet” website (www.findavet.rcvs.org.uk, RCVS, London, UK).

### 2.2. Questionnaire Structure

The questionnaire was developed in Survey Monkey (SurveyMonkey Inc, Palo Alto, CA, USA; www.surveymonkey.com). The questionnaire consisted of four main sections and used a mixture of open questions, closed questions, multiple choice and opinion questions requiring a response on a Likert scale. Section one gathered background information about the respondents. The second section asked for specific information relating to current knowledge of, and past education on, clinical audit. Section three considered the experience the respondent already had of clinical audit, either as a participant in a clinical audit set up by another individual and/or as an individual that had set up a clinical audit themselves. The fourth section asked for current attitudes towards clinical audit and used some questions based on those found in publications by Viner [5,12]. Skip logic (a function that allows the respondent to skip to a future question based on the answer choice selected) was used throughout the questionnaire to avoid participants responding to questions not applicable to them [13]. Questions with skip logic required an absolute answer while all other questions were optional. A copy of the questionnaire can be found in the Supplementary File 1.

### 2.3. Questionnaire Development and Distribution

The questionnaire was constructed and then pre-tested and piloted by members of the Centre for Evidence-based Veterinary Medicine (CEVM) at the University of Nottingham and nine practising farm animal veterinary surgeons. The questions were altered based on the trial respondent’s feedback.

The questionnaire was distributed by the Sheep Veterinary Society (SVS) to its members via an email containing the survey link on 27 July 2015. A website and a quick response (QR) code link to the questionnaire was included in the July 2015 BCVA newsletter (posted to members in paper format, electronically by email and available as a PDF online and featured on the BCVA website).

The RCVS “Find a Vet” website advanced search (http://findavet.rcvs.org.uk/find-a-vet/advanced-search/, RCVS, London, UK) was used to find email addresses for veterinary practices listed as providing veterinary services for sheep and cattle. The search found 842 practices. Practices that did not have email addresses and practices whose email address suggested they did not provide farm animal services (for example dogsandcats@avetpractice.com) were removed from the list, resulting in 567 relevant email addresses. An email inviting participation from farm animal veterinary surgeons was sent to these 567 email addresses on 29 July 2015, with a reminder sent four weeks and six weeks later.

The questionnaire was also distributed sporadically during the two-month period via social media; specifically via the personal Facebook and Twitter accounts of the author and the CEVM. A link to the questionnaire was also made available on the CEVM website. Respondents completing the questionnaire were offered the chance to enter a prize draw to win £50 worth of vouchers from a store of their choice. The questionnaire was closed and no longer available to participants on 23 September 2015.

### 2.4. Data Management and Analysis

Responses received from Survey Monkey were downloaded into a Microsoft Excel V.14.0.6 file (2010 Microsoft Corporation, Redmond, DC, USA). The dataset was managed, analysed statistically and tables and figures generated using Microsoft Excel V.14.0.6.

The results were analysed using both qualitative and quantitative methods. Pearson’s Chi-squared tests were used to assess statistical associations between whether the participants undertook clinical audit compared with the respondents’ age, clinical audit education, work pattern and caseload. Statistical significance was set at *p* < 0.0125 after applying a Bonferroni correction for multiple testing [14]. A Pearson’s Chi-squared test was also used to determine if there was a difference in undergraduate education provision between veterinary surgeons qualifying pre- and post-2010. Statistical significance for this was set at *p* < 0.05.

Responses to the free text answers were analysed in Microsoft Excel V.14.0.6 using inductive thematic analysis [15]. This is a process whereby commonalities or patterns between responses given by participants are identified and grouped into overarching themes. The questions analysed using thematic analysis focused on the opinions of the respondents about their prior experiences, what was important to them about the clinical audit process and any advice that respondents would pass on to other vets attempting clinical audit for the first time.

### 2.5. Ethical Approval and Data Protection

This project received ethical approval from the ethics research committee at the School of Veterinary Medicine and Science at The University of Nottingham (Ethical approval number 1453 150707). Email addresses entered for the prize draw were separated from the questionnaire responses prior to commencement of data analysis. These email addresses were permanently deleted once the prize had been allocated.

## 3. Results

Three hundred and thirty-two responses were collected across the eight-week study period. Not all respondents answered every question (denominators are given where required for each question), however the majority of people (*n* = 316/332; 95%) entered all or some information through to the final section.

### 3.1. Background Information about the Participants

Forty-five percent (*n* = 148/332) of the veterinary surgeons that participated carried out solely farm animal clinical work while 54% (*n* = 180/332) undertook a mixed clinical case load. The majority of the respondents worked in clinical practice full time (92%; *n* = 296/323), compared to part time (8%; *n* = 27/323). The year of graduation of the participants ranged from 1975–2015, with 49% (*n* = 155/317) being classified as “recent graduates” (less than eight years qualified) by the British Veterinary Association [16].

### 3.2. Current Knowledge of and Past Education on Clinical Audit

A large proportion (*n* = 237/325; 73%) of respondents had heard of clinical audit previously, whilst *n* = 79/325 (25%) had not, and *n* = 9/325 were unsure if they had (3%). Two hundred and nineteen veterinary surgeons provided a definition of clinical audit. The majority of the respondents used the words “assess”, “review”, “evaluate” or “analyse” in the definition they provided (Table 1). Infrequently, negative definitions of clinical audit were suggested including “being checked up on by a third party”, “having my work investigated” and “caseload accounting”.

Less than one-fifth (*n* = 31/165; 19%) of the veterinary surgeons reported receiving training on clinical audit at veterinary school while twenty-two percent (*n* = 47/217) recalled undertaking postgraduate training on the subject (Table 2). Significantly more veterinary surgeons who had graduated after 2010 reported receiving undergraduate training on clinical audit compared to clinicians graduating in previous decades (*p* < 0.001).

### 3.3. Experience of Clinical Audit

Forty-nine percent of respondents (*n* = 148/301) stated that they had not carried out clinical audit in any species, while 48% (*n* = 143/301) of respondents nominated that they had carried out clinical audit in any species (10 people did not know). Participants who recalled receiving postgraduate training in clinical audit were significantly more likely to report that they had conducted an audit in any species than those who had not (*p* = 0.008). Working pattern (full or part time; *p* = 0.26), caseload (farm animal vs mixed; *p* = 0.07) and year of graduation (pre-2010 vs. 2010 onwards; *p* = 0.21) had no significant association with whether respondents had or had not previously conducted a clinical audit in any species. Of the people that said they had carried out a clinical audit in any species (*n* = 143), 126 respondents (*n* = 126/143; 88%) indicated that they had previously been involved in a clinical audit specifically in farm animal veterinary practice.

### 3.4. Most Recent Clinical Audit

**Figure 1:**
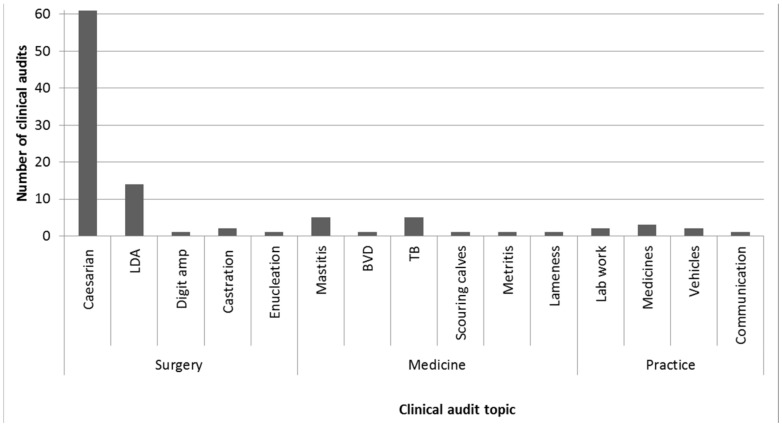
The topic of the most recent clinical audit conducted by the questionnaire respondents in a survey investigating the experiences and attitudes of UK farm animal clinicians towards clinical audit (*n* = 101). LDA = left displaced abomasum, Digit amp = digit amputation, BVD = bovine viral diarrhoea, TB = bovine tuberculosis.

One hundred and twenty-four participants described why the clinical audit they had most recently been involved with had been undertaken; 58 people gave more than one reason resulting in a total of 184 listed reasons. The most common reason was to gather information on what happens in the practice (*n* = 86/184; 48%), to meet RCVS Practice Standards Scheme requirements (*n* = 41/184; 22%), in response to a recent significant event (*n* = 25/184; 14%) and to meet RCVS Continuing Professional Development requirements (*n* = 6/184; 3%). Seventy-eight percent (*n* = 79/101) of the topics of the most recently conducted audits were surgical, with the majority of these (*n* = 61/79; 77%) focusing on caesarean sections (Figure 1).

Reasons given as to why these particular topics had been chosen were varied (*n* = 114). Many of the respondents highlighted reasons such as to determine what was currently being done in their practice and a few felt the topic chosen was considered easy to do and the data easy to collect. Other reasons given were to standardise the practice’s approach to cases, to improve services and in response to unexpected outcomes.

### 3.5. Experience of Coordinating a Alinical Audit

Twenty-five respondents indicated that they had set up and run their own clinical audit in farm animal practice. Of these, nearly half stated they had completed all the clinical audits that they had set up (*n* = 11/25; 44%) while over half (*n* = 14/25; 56%) stated they had started but not finished at least one clinical audit at the time of the survey. Of the 25 respondents who answered subsequent questions about setting up their own audit, eight (*n* = 8/23; 35%) of these individuals advised that they had received training in clinical audit at a postgraduate level and two (*n* = 2/17; 12%) recalled receiving training during their undergraduate degree.

When using standards as part of their own clinical audits (*n* = 25; a total of 35 responses were given to the question), nine participants reported using standards found in the literature (*n* = 9/35; 26%), nine described the use of pre-existing local standards such as protocols or guidelines (*n* = 9/35; 26%) and nine reported they had used standards created within the practice for the purpose of the audit (*n* = 9/35; 26%). Four veterinary surgeons advised they had used standards gained after running the first round of audit (*n* = 4/35; 11%), and four veterinary surgeons did not recall using any standards (*n* = 4/35; 11%).

Five overarching themes were identified from 19 comments in relation to advice veterinary surgeons who had coordinated their own clinical audits would give those setting up and running one for the first time (Table 3). Many respondents stressed the importance of good preparation.

### 3.6. Experience of Participating in a Clinical Audit

**Figure 2:**
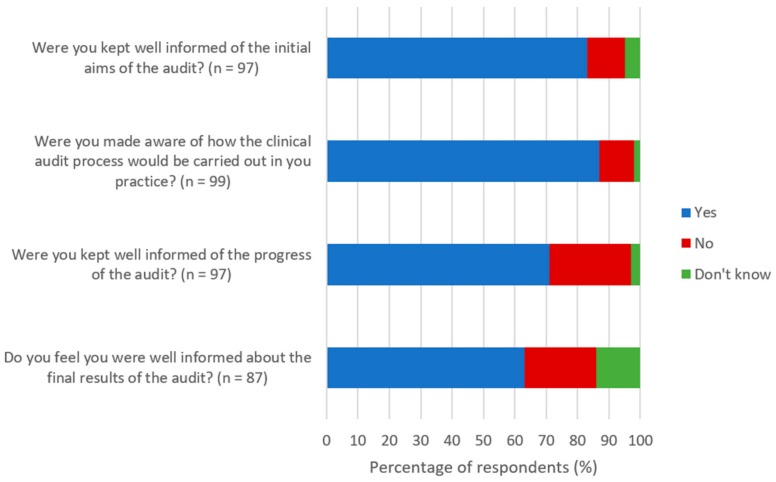
How well informed the participants felt they were kept about the most recent clinical audit conducted in their practice. Taken from a survey investigating the experiences and attitudes of UK farm animal clinicians towards clinical audit.

Ninety-five participants (*n* = 95/124; 77%) indicated that they had participated in, but had never set up and run their own clinical audit in farm animal practice. Over 60% of the respondents that were asked about their level of involvement in the most recent clinical audit reported to be happy that they were kept well informed about the audit that they were participating in (Figure 2).

The overarching themes and subthemes that were identified from the 61 comments for advice that respondents would give to other veterinary surgeons who were participating in clinical audits are shown in Table 4. Many of the respondents mentioned the importance of good communication in their comments. This included clear communication of initial aims, ongoing progress and results of the audit, as well as circulating information and holding meetings to ensure all audit members stay informed and felt involved in the process.

Individual and team participation was highlighted as important parts of the audit process by some of the participants. Some veterinary surgeons suggested that the hard work was worth the effort with much to learn about yourself and others. Many of the comments mentioned how audit data was collected and handled as well as the overall quality of the information. Some of the vets highlighted the need for easy and fast data collection methods with the use of concise questions. A simple, realistic and well organised audit with defined targets and some background research on the topic were highlighted as important factors in a successful audit.

### 3.7. Attitudes towards Clinical Audit

**Figure 3:**
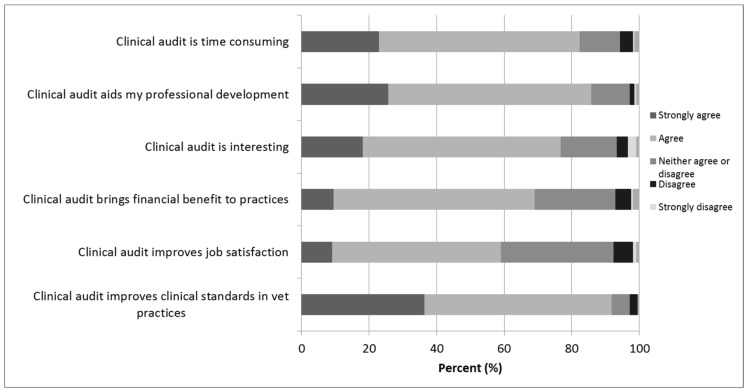
Attitudes towards clinical audit from veterinary surgeons that had heard of clinical audit before gave in a survey investigating the experiences and attitudes of UK farm animal clinicians towards clinical audit (*n* = 210). Questions from Viner (2003) and (2006).

Attitudes towards clinical audit appeared to be generally positive (Figure 3). More than three quarters of respondents agreed with statements that clinical audit improved clinical standards in veterinary practice, aided their own professional development and that the process was interesting. There was slightly less agreement with the statement that clinical audit improved job satisfaction, with 33% (*n* = 70/210) neither agreeing nor disagreeing, and 83% (*n* = 173/210) of participants agreeing with a statement that the process was time consuming.

### 3.8. Final Comments

The participants were asked for any further comments they might have about clinical audit in farm animal practice. The 26 comments provided are summarised in Table S1. The respondents generally agreed that clinical audit was a beneficial tool to use in practice, but that it required time to undertake and needed to be conducted properly for benefits to be realised.

## 4. Discussion

This unique study, the first to focus on the experiences of farm animal veterinary surgeons, has found that there is substantial variation in the use and understanding of clinical audit. However, general attitudes towards the topic appear to be positive despite just under 50% having experience with clinical audit. The results of this study can aid the development of clinical audit guidance, as well as informing future policy on the topic to ensure continued appropriate levels of client and patient care.

In agreement with the situation observed by Grol and Wensing [10] in the medical field, not all practitioners appear to have implemented the process, and a larger proportion of farm animal veterinary surgeons (48% (*n* = 143/301)) appear to be conducting clinical audit in practice in 2015 compared to the 18% of 904 practitioners that participated in the study by Viner [5]. However, just 4% of the survey sample in Viner’s study did not understand the concept of clinical audit while, in the current study, 24% had not heard of the concept. This may be because a definition was provided to participants by Viner [5], and in the current study one was deliberately not given in order to gain more information from the answers about the respondents’ knowledge of the topic. Response bias may also have played a part in either of these results; individuals may feel inclined to provide an untrue answer when asked about something they think they should be doing as part of their professional responsibilities [17]. The difference could also be due to the population surveyed with the current research focussing specifically on farm animal veterinary surgeons and differences in the way that questions were asked.

Despite clinical audit being mentioned in the RCVS Code of Conduct, the Practice Standards Scheme, recent presentations at CPD events and some publications, these results suggest that more needs to be done by all stakeholders to reach veterinary surgeons in practice to advise them on what clinical audit is and how to use it. Post-2010 graduates were found to be significantly more likely to have received education on clinical audit whilst at veterinary school. This may be because clinical audit was introduced into the curriculum at that time, or these responses could have been influenced by recall bias. The results suggest that there may be a lack of knowledge in veterinary surgeons who graduated prior to 2010 who may require extra support with the process. Waine and Brennan [3] suggest some of the confusion surrounding what clinical audit is may stem from the description of different types of audit, and a simple definition of clinical audit itself may make the process more user-friendly in the future.

The reasons given by respondents as to why they conducted their most recent clinical audit are insightful and demonstrate that clinical audit is carried out for motives far beyond meeting RCVS requirements. It appears that vets are primarily focused on using clinical audit to determine what happens in their practices, what the outcome of their cases are and where services can be improved. This highlights the benefits that clinical audit can have as a method of further incorporating the principles of evidence-based medicine into practice by focusing on improving clinical decision-making. Additionally, in agreement with previous studies [5,12], veterinary attitudes towards clinical audit are generally positive, with benefits nominated such as improved clinical standards, professional development and to some extent improved job satisfaction, which is a more personal motivation. These are seemingly more positive than the attitudes held by human medics [8,18] where clinical audit is often conducted to ensure that the correct procedures are being conducted. This is perhaps easier in a centralised system with a single governing entity like the National Health Service (NHS) in the UK, which is different to veterinary practice. In some circumstances, there are legitimate reasons where staff should be monitored, but this must be handled in an appropriate way to avoid staff feeling they are ‘being checked up on’ and not trusted.

Veterinary surgeons appear to have conducted clinical audit on a wide range of topics, with surgical topics being the most popular in the recent audits conducted. These appear to have several advantages such as being easily definable cases with good caseloads and clear outcomes. It should be noted that at the time the survey was conducted, XL Vets (www.xlvets.co.uk, XLvet UK Ltd., London, UK), a cooperative of over 50 UK farm animal businesses, was running a nationwide clinical audit of cattle caesarean sections. This could have impacted the results here; at the last report, over 4000 caesareans had been recorded, suggesting a very high participation rate either by individuals or geographically, or both. It is not known how the XL Vets clinical audit may have impacted on the views, opinions, attitudes or the experiences of the veterinary surgeons responding to this survey.

Cai, Greenall, Cai and Ding [18] conducted a survey of junior doctors and found that they were unsure of the definition of clinical audit, often failed to complete audits, and that team members needed to work together in ‘office hours’ to conduct an effective clinical audit. It has also been suggested that time to undertake a clinical audit is a barrier [8]. Many of these barriers have also been highlighted by veterinary surgeons in the current study and support previous findings by others [5]. It is clear that providing protected time to conduct the process effectively is crucial and should be considered by practice managers. Additionally, good communication and preparation appears to be key to running successful clinical audits in veterinary practice.

### Study Limitations

The questionnaire was created and delivered electronically. Veterinary surgeons who do not use the internet and who are not members of BCVA or the SVS will not have received the survey. This creates some respondent bias, however responses were received from individuals from a variety of different age groups working in a variety of different types of practice and respondents were also given the opportunity to participate via the RCVS practice list, and social media, which goes someway to negating this. People more interested in clinical audit, for positive or negative reasons, may have been more likely to respond to the questionnaire than those who were less interested. Although email addresses entered for the prize draw were removed from the data before analysis took place, individuals that did provide an email address may have provided different responses had the survey been truly anonymous.

This survey did not account for clinical audits that were still currently running at the time the participants completed the survey. This may have affected some of the results where candidates were asked about completion rates or being informed about the whole process.

Thematic analysis has a number of limitations [15] and the analysis was carried out by just one individual. It is possible that another individual may have interpreted the data in an alternate way. Additionally, the results here relate to responses nominated by veterinary surgeons and it is unknown how these responses relate to actual activities undertaken.

## 5. Conclusions

This study highlights for the first time the range of understanding, experience and use of clinical audit by farm animal veterinary surgeons in UK veterinary practice. The results suggest that clinical audit is a tool that is, and can be, used with success in the farm animal practice setting and more importantly, is a process that is currently seen as positive by the majority of the farm animal veterinary profession. There are some associated challenges with the process, with allocated time to run clinical audits one of the main barriers. It is apparent that future work should focus on ways of encouraging the profession to conduct clinical audit, and the requirement for further development of education programmes, resources and support for practitioners.

## Figures and Tables

**Table 1:** The themes that were identified from the suggested definitions of clinical audit by questionnaire respondents in a survey investigating the experiences and attitudes of UK farm animal clinicians towards clinical audit (*n* = 219).

Description of Themes	Example Quotes
to review/assess/analyse/appraise	“To review cases and their results” “Examining practices and related data to determine success rates and evaluate performance” “Analyse and assess successes and failures in clinical treatment of cases/situations”; “… also assess and appraise treatment regimes …”
finding the best way to do things	“Gathering information on what is done and reviewing this information to try to find best practices”
improving clinical procedures or outcomes for the future	“Monitoring clinical outcomes of actions/treatments in different situations to assess success/failure & hence modify future actions/treatments”
to check ‘best practice’ is being delivered	“An assessment of one’s clinical skills to check they are still up to date and offering ‘best practice”
monitoring against a standard	“Reviewing procedures and practices against ‘best practice’ standards”
peer review	“Other veterinarians checking work performed to see if of acceptable standard-peer review if you like”
bench marking	“System for monitoring clinical performance within practice and possible benchmarking or a feedback system”;
protocols	“A system where for example morbidity/mortality is recorded, data assessed, outcomes discussed and where necessary changes in procedures and protocols established” “A critical review of treatment protocols”; “Checking if the protocols you follow are correct and work”; “A structured quality assurance protocol that aims to improve patient care and clinical outcomes ”

**Table 2:** The proportion of respondents who nominated that they had received undergraduate and/or postgraduate training on clinical audit by decade of graduation in a survey investigating the experiences and attitudes of UK farm animal clinicians towards clinical audit. Table includes responses from individuals answering questions both on year of graduation and undergraduate (*n* = 159)/postgraduate education (*n* = 209).

Decade of Graduation	Undergraduate Education				Postgraduate Education			
	*n*=	Yes	No	Don’t Know	*n*=	Yes	No	Don’t Know
1970s	9	0 (0%)	9 (100%)	0 (0%)	10	1 (10%)	9 (90%)	0 (0%)
1980s	15	0 (0%)	15 (100%)	0 (0%)	20	4 (20%)	15 (75%)	1 (5%)
1990s	29	1 (3%)	27 (94%)	1 (3%)	34	4 (12%)	29 (85%)	1 (3%)
2000s	55	2 (4%)	50 (91%)	3 (5%)	80	20 (25%)	59 (74%)	1 (1%)
2010s	51	20 (39%)	26 (51%)	5 (10%)	65	18 (28%)	45 (69%)	2 (3%)
Total:	159				209			

**Table 3:** Main themes identified from advice that veterinary surgeons who have set up and run clinical audits previously nominated they would give to other veterinary surgeons wishing to set up clinical audits. Taken from a survey investigating the experiences and attitudes of UK farm animal clinicians towards clinical audit.

Theme	Subtheme	Example Quote
audit preparation	defining aims, purpose and outcomes	“Have a specific aim that you want to achieve with clinical audit (e.g., unified practice policy on LDA correction technique), and make sure everyone involved understands and agrees with that aim before commencing audit”
	choosing the topic	“Research the topic and case prior to discussion”
	setting a time limit	“Include a specified time period”
	literature	“Read the literature, try to decide if there is a ‘gold standard’ treatment or method for the procedure/condition, or if there are any methods/treatments that are no longer appropriate”; “Gain access to a full reference library, many articles necessary for determining best practice are not freely available. Learn how to review published literature effectively, often papers misinterpret the evidence they present, often favourably!”
communication	importance of good communication	“… involve as many stakeholders within the audit as possible and communicate well as to the purpose of the audit …”
	existing communication	“If you communicate with staff and clients you should not need one, its common sense and unnecessary!”
data	collection methods	“Threaten anybody who doesn’t fill the form, collect ear numbers etc. so that you can get full data!”
	detail	“… relies on vets recording data accurately …”
	software systems	“Learn how to use excel spreadsheets”
motivational tips	for individuals	“Do it-you might surprise yourself”
	for the team	“… it is well worth it”
negative audit experiences	participation	“Be prepared for arguments”
	resources	“It is not easy, and difficult to get records and get busy people to participate”

**Table 4:** Advice from veterinary surgeons who have participated in clinical audit for veterinary surgeons who are about to participate in a clinical audit set up by somebody else. Taken from a survey investigating the experiences and attitudes of UK farm animal clinicians towards clinical audit.

Theme	Subtheme	Example Quote
communication	audit purpose	“Ensure you understand the aims of the clinical audit, and exactly what data needs to be collected”
	audit process	“Communication of progress and results essential”; “Constant communication is essential and results need to be made readily available”
participation	individual	“Get involved early to make sure outcomes are agreed”
data	quality	“That the info gathered will help direct future policy, avoid litigation, improve standard of treatment—therefore answer as truthfully as possible”; “Give accurate answers and feedback—positive and negative”
	collection	“Make time and make the questions easy and fast …”
audit process	time	“It takes time to do so make sure it has been thought through”
	preparation	“Getting someone enthusiastic and who knows what they are doing to run it”

## Supplementary Materials

The following are available online at http://www.mdpi.com/2306-7381/5/4/84/s1, Supplementary File 1: Questionnaire used during a survey investigating the experiences and attitudes of UK farm animal clinicians towards clinical audit (see accompanying pdf); Table S1: Final comments from the participants of the questionnaire on clinical audit.
