# Supplementary material for: A Cross-Sectional Study of Experiences and Attitudes towards Clinical Audit of Farm Animal Veterinary Surgeons in the United Kingdom
Source: Vet Sci. 2018 Sep 28;5(4):84. doi: 10.3390/vetsci5040084 (PMC6313848; doi:10.3390/vetsci5040084)
Supplement: Supplementary file 1 [file vetsci-05-00084-s001.zip › Suppl/vetsci-347434-supplementary-english.docx]

**Table S1.** Final comments from the participants of the questionnaire on clinical audit (CA).

| Theme | Subtheme | Example Quote |
| --- | --- | --- |
| audit resources | time | “Having been involved with clinical audit, I feel it’s a really worthwhile task but does require the time to do it” |
|  | protocols | “Audit leads to protocol, and devising protocols that enhance clinical care whilst not restricting the practitioner is very challenging” |
| benefits of audit | clinical standards | “CA may improve or may not improve standards in veterinary practice” |
|  | CPD | “But handled properly can be fun, part of CPD, and teach everyone something” |
|  | new graduates | “I think as a new graduate clinical audit could be very valuable to assess both your own performance, and gather information from other practitioners to learn and improve” |
|  | education | “Much of on-farm practise is solo vet charge with no objective criticism unless discussed informally back at the practice. Objective criticism of clinical procedures/outcomes should improve efficiency and learning” |
| barriers to audit | time | “Would like to carry out a clinical audit but time constraints are the main barrier” |
|  | defining cases or outcomes | “… it depends on how the outcomes are measured.  Success is a subjective word and needs defining!” |
|  | topic | “It is interesting depending on the topic being audited!” |
|  | communicating results | “It is an interesting exercise but difficult to communicate externally” |
|  | education | “More should be done at uni level to inform students”; “More teaching should be done at vet school”. |
| motivators to audit | personal | “Gives a guide to your personal competence and success rates” |
|  | benefits | “But handled properly can be fun, part of CPD, and teach everyone something” |
