# Supplementary material for: A Cross-Sectional Study of Experiences and Attitudes towards Clinical Audit of Farm Animal Veterinary Surgeons in the United Kingdom
Source: Vet Sci. 2018 Sep 28;5(4):84. doi: 10.3390/vetsci5040084 (PMC6313848; doi:10.3390/vetsci5040084)
Supplement: Supplementary file 1 [file vetsci-05-00084-s001.zip › Suppl/vetsci-347434-supplementary.pdf]

Experiences and attitudes of UK farm animal veterinary surgeons towards

Thank you for taking part in our study.

We would like to invite you to take part in a study organized by the Centre for Evidence-based Veterinary Medicine (CEVM) at the School of Veterinary Medicine and Science, University of Nottingham.

We are conducting a survey of farm animal veterinary surgeons in the UK to collect information on experiences and attitudes towards clinical audit. The information gathered will assist the development of guidelines for farm animal veterinary surgeons on how to carry out clinical audit in practice.

All farm animal veterinary surgeons who are members of the British Cattle Veterinary Association or the Sheep Veterinary Association have been invited to take part in this study. All practices listed on the RCVS register as offering farm animal veterinary services have also been invited to participate.

Your participation in this research in entirely voluntary. There is no obligation to take part although your responses will be of immense benefit to us. There is no payment for taking part in this study, however if you choose to submit your email address you will be entered into a prize draw to win £50 worth of vouchers for a shop of your choice. By completing and submitting the questionnaire online you are indicating your consent to participate in the study. The study is likely to take between 5 and 10 minutes depending on what questions are appropriate to you. If you do not wish to participate in this study, please exit the webpage now.

All information collected will be stored safely, treated in the strictest confidence and fully anonymised; no individual person will be identifiable in any publication. Only researchers at the CEVM will have access to your completed questionnaire. The findings of this research will be published in peer-reviewed scientific journals and presented at conferences. Summary findings will be available to participants on request.

At the end of the questionnaire you are invited to submit your email address to enter the prize draw. Your email address will be removed from the rest of your responses so that individual answers can not be tracked back to you. Your email address will also not be passed on to anyone.

The CEVM is sponsored by the University of Nottingham and Elanco Animal Health. These organisations have had no input into the study design of the questionnaire and will not have access to the unpublished data. This research has received ethical approval from the School of Veterinary Medicine and Science Ethics Committee, The University of Nottingham

If you would like more information about the study, the Centre for Evidence-based Veterinary Medicine, or have any other questions please contact Katie Waine at svxkw@nottingham.ac.uk or Marnie Brennan on 0115 951 6577 (phone), or email marnie.brennan@nottingham.ac.uk

We would be delighted if you would complete the questionnaire. Your help is very important to the success of this study, so we appreciate your time and co-operation.

Many thanks in anticipation of your help.

Katie Waine BVSc MRCVS PhD Student
Marnie Brennan BSc(VB) NVMS PhD MRCVS, Deputy Director of CEVM

Background information

Are you a veterinary surgeon undertaking farm animal clinical work, on farm, in the United Kingdom?
('Farm animal' includes cattle, sheep, goats, pigs and poultry. 'Clinical work' involves the examination, diagnosis and treatment of farm animals as an MRCVS. 'On farm' means that for the majority of the time you treat these animals at their holding, as opposed to in the consult room at the veterinary practice.)

- ◯ Yes
- ◯ No

Where did you hear about this study? (please tick all that apply)

- ☐ I received an email from svxkw@nottingham.ac.uk
- ☐ I received an email from the Britich Cattle Veterinary Association (BCVA)
- ☐ I received an email from the Sheep Veterinary Society (SVS)
- ☐ Twitter
- ☐ Facebook

Other (please specify)

Do you work as a practising veterinary surgeon on a full time or part time basis?

- ◯ Full time
- ◯ Part time

Please tick the box that most accurately describes the work that you undertake when working as a practising veterinary surgeon:

- ☐ I work in mixed veterinary practice (Small and farm animals only)
- ☐ I work in mixed veterinary practice (Small, farm and equine)
- ☐ I work in large animal practice only (Equine and farm)
- ☐ I work in farm animal practice only (Farm only)

Other (please specify)

What year did you graduate with your veterinary degree?

Clinical audit

Had you heard of clinical audit before receiving this questionnaire?

- ◯ Yes
- ◯ No
- ◯ I don't know

Please define what you think the term 'clinical audit' means in your own words:

Did you receive any undergraduate training on clinical audit?

- ◯ Yes
- ◯ No
- ◯ Don't know

Have you received any postgraduate training on clinical audit, for example as part of Continued Professional Development (CPD)?

- ◯ Yes
- ◯ No
- ◯ Don't know

Experience of clinical audit

Clinical audit is the review and improvement of clinical care provided to patients.

Have you ever been involved in a clinical audit within farm animal veterinary practice?

- ◯ Yes
- ◯ No
- ◯ I don't know

Experience of clinical audit

Please answer the following two questions in relation to the last clinical audit that you were involved with.

What was the topic of the audit?

Why was this topic to audit chosen?

Experience of clinical audit

Please indicate your involvement with clinical audit in farm animal practice:

- ◯ I have set up and run clinical audit(s) in farm animal practice
- ◯ I have participated in clinical audits but i have never set up and run my own clinical audit
- ◯ I don't know

Experience of clinical audit

These questions relate to individuals who have set up and run clinical audits in farm animal practice.

Clinical audit involves four main steps:
Stage 1: Preparation and planning
Stage 2: Measuring performance
Stage 3: Making and implementing changes
Stage 4: Sustaining improvement through re-audit

Based on a full clinical audit including all four steps listed above, how many clinical audits have you completed?

Based on a full clinical audit including all four steps listed above, how many clinical audits have you started but not completed?

Often in clinical audit, standards are used at the beginning of the audit process to determine if there is any guidance on best practice, or at the end of the audit to compare the audit results to. Thinking of your most recent clinical audit that you ran, did you use any standards in any part of the audit process? (Please tick all that apply)

- ☐ I used standards found in the published literature
- ☐ I used pre-existing local standards (for example practice guidelines or protocols)
- ☐ Standards were created within the practice for the purpose of the audit
- ☐ I ran one loop of the audit and used the initial results to create my own standard
- ☐ No standards were used
- ☐ I don't know

What three pieces of advice would you give to somebody who is about to set up a farm animal clinical audit for the first time?

1.

2.

3.

Additional to setting up your own clinical audit, have you ever participated in a clinical audit that was set up by somebody else?

- Yes
- No
- I don't know

Experience of clinical audit

These questions relate to individuals who have participated in farm animal clinical audit, but where they did not set up and run the audit themselves. Participants may have been involved with various stages of the audit such as collecting data or choosing a topic to audit.

How many clinical audits have you participated in that have been set up by someone else in farm animal practice?

Thinking about the last clinical audit that you participated in, do you feel that you were kept well informed of the initial aims of the audit?

- Yes
- No
- I don't know

Please enter any additional comments here:

Thinking about the last clinical audit that you participated in, were you made aware of how the steps of the audit process would work in your practice?

- Yes
- No
- I don't know

Please enter any additional comments here:

Thinking about the last clinical audit that you participated in, were you kept well informed of the progress of the audit?

- ◯ Yes
- ◯ No
- ◯ I don't know

Please enter any additional comments here:

Thinking about the last clinical audit that you participated in, do you feel that you were well informed about the final results of the audit?

- ◯ Yes
- ◯ No
- ◯ I don't know

Please enter any additional comments here:

What three pieces of advice would you give to someone participating in a farm animal clinical audit that someone else set up?

1.

2.

3.

Attitudes towards clinical audit

Please rate your level of agreement with the following statements (Where 1 = strongly agree, and 5 = strongly disagree)

	Strongly agree	Agree	No opinion	Disagree	Strongly disagree	I don't know
I think clinical audit can improve clinical standards	◯	◯	◯	◯	◯	◯
I think clinical audit can improve job satisfaction	◯	◯	◯	◯	◯	◯
I think clinical audit can bring financial benefit to veterinary practice	◯	◯	◯	◯	◯	◯
I think clinical audit is interesting	◯	◯	◯	◯	◯	◯
I think clinical audit can be time consuming	◯	◯	◯	◯	◯	◯
I think clinical audit aids my own clinical development	◯	◯	◯	◯	◯	◯

Please include any further comments that you may have about clinical audit in farm animal practice here:

Thank you!

Thank you for completing our survey!

If you would like to be entered in to the prize draw to win £50 worth of vouchers for a shop of your choice, or if you would like to be informed about the results of the study, please enter your email below and tick the relevant box(es). Your email address will not be used for any other purpose, nor will it be passed on to any third parties. If you do not wish to be informed of the results, or to be entered into the prize draw, please leave blank.

☐ Yes, I would like to be entered into the prize draw to win £50 worth of vouchers for a shop of my choice (the winner will be notified via email)

☐ Yes, I would like to be informed about the results of this study via email

Please enter you email address here:
